# The African Region early experience with structures for the verification of measles elimination – a review

**DOI:** 10.11604/pamj.supp.2020.35.1.19061

**Published:** 2020-01-02

**Authors:** Balcha Masresha, Richard Luce, Patricia Tanifum, Emmaculate Lebo, Annick Dosseh, Richard Mihigo

**Affiliations:** 1 WHO Regional Office for Africa, Brazzaville, Congo; 2 WHO, Inter-country team for Western Africa, Ouagadougou, Burkina Faso; 3 WHO, Inter-country team for Central Africa, Libreville, Gabon; 4 WHO, Inter-country team for Eastern and Southern Africa, Harare, Zimbabwe

**Keywords:** Measles elimination, verification, Africa

## Abstract

Substantial progress has been achieved in the last two decades with the implementation of measles control strategies in the African Region. Elimination of measles is defined as the absence of endemic transmission in a defined geographical region or country for at least 12 months, as documented by a well-performing surveillance system. The framework for documenting elimination outlines five lines of evidence that should be utilized in documenting and assessing progress towards measles elimination. In March 2017, the WHO regional office for Africa developed and disseminated regional guidelines for the verification of measles elimination. As of May 2019, fourteen countries in the African Region have established national verification committees and 8 of these have begun to document progress toward measles elimination. Inadequate awareness, concerns about multiple technical committees for immunization work, inadequate funding and human resources, as well as gaps in data quality and in the implementation of measles elimination strategies have been challenges that hindered the establishment and documentation of progress by national verification committees. We recommend continuous capacity building and advocacy, technical assistance and networking to improve the work around the documentation of country progress towards measles elimination in the African Region.

## Introduction

The WHO global vaccine action plan 2011-2020 outlines a goal for the elimination of measles and rubella in at least 5 WHO regions by 2020 [1]. In the African region, the regional goal for measles elimination was adopted in 2011, with a target date for 2020 [2]. The regional strategies to achieve elimination include increasing access and coverage with routine immunization services in all districts; achieving high coverage during all measles Supplemental Immunization Activities (SIAs), as well as improving the quality of measles surveillance. The member states adopted a goal with the following targets: i) ≥ 95% coverage with the first dose of measles-containing vaccine (MCV1) at national and district levels; ii) ≥ 95% coverage in all districts during measles SIAs; iii) confirmed measles incidence <1 per million population in all countries; iv) Attaining high quality measles surveillance as evidenced by a non-measles febrile rash illness (NMFRI) ≥ 2 per 100,000 population annually and the collection of a blood specimen from at least 1 suspected measles case in at least 80% districts annually [2, 3]. As of April 2019, the African Region does not yet have a goal targeting rubella/Congenital Rubella Syndrome (CRS) elimination. However, countries in the region are using the opportunity of implementation of measles elimination strategies to introduce rubella vaccine and to document the epidemiology of rubella through the existing measles case based and lab-supported surveillance system. By the end of April 2019, a total of 29 of the 47 countries in the region have introduced rubella vaccine in their vaccination schedules [4]. Currently, only a limited number of countries have implemented sentinel surveillance and/or retrospective reviews of clinical records for Congenital Rubella Syndrome (CRS) [5]. Substantial progress has been achieved in the last two decades with the implementation of measles control strategies in the African Region. By the end of 2017, 8 (17%) of the 47 countries have coverage ≥ 95% according to the WHO-UNICEF estimates for national MCV1 coverage; 32 (74%) of 43 countries attained ≥ 95% administrative coverage in their most recent measles or measles-rubella SIAs; 23 (52%) of 44 countries in the case-based surveillance network have met the targets for the two principal surveillance performance indicators. Reported incidence of confirmed measles is less than 1 per million population in 20 (45%) of the 44 countries reporting case-based surveillance data regularly [4]. Between 2000 and 2017, estimated measles mortality declined by 86% in the African Region of the WHO [6].

## The framework for verification of measles elimination

Elimination of measles is defined as the absence of endemic transmission in a defined geographical region or country for at least 12 months, as documented by a well-performing surveillance system. The 3 criteria for verifying measles and rubella elimination include: i) the documentation of the interruption of endemic measles and rubella virus transmission for a period of at least 36 months from the last known endemic case; ii) the presence of a high-quality surveillance system; iii) measles virus genotyping information that supports interruption of endemic transmission [7, 8]. The global and regional frameworks for the verification of measles elimination require that countries establish independent structures charged with compiling the programmatic and epidemiological information necessary to assess progress and document measles elimination [8]. This includes the establishment of National Verification Committees (NVC) with the primary responsibility for guiding countries in the preparation of their documentation of progress towards the achievement of measles elimination, as well as the Regional Verification Commission (RVC), which validates and verifies elimination in each country and eventually in the Region.

The framework for documenting elimination outlines five lines of evidence that should be utilized in documenting and assessing progress towards measles elimination: 1) a detailed description of the epidemiology of measles and rubella since the introduction of measles and rubella vaccine in the national immunization program; 2) population immunity, presented as a birth cohort analysis with the addition of evidence related to any marginalized and migrant groups; 3) quality of epidemiological and laboratory surveillance systems for measles and rubella; 4) sustainability of the national immunization program, including resources for interventions to sustain elimination; 5) genotyping evidence that measles and rubella virus transmission has been interrupted [7, 8].

When evaluating the lines of evidence, NVCs and RVCs are expected to review all the available data at both national and subnational levels that can be assessed to determine whether elimination has been achieved. The five lines of evidence facilitate a comprehensive evidence-based assessment of population immunity at all levels, immunization program performance and the capacity to sustain elimination.

The WHO African regional standards for case-based measles surveillance have been in place since 2004, with an update in 2015 to include an optional elimination-standard surveillance which is recommended for countries with confirmed measles incidence approaching or less than 1 per million population. Elimination standard surveillance is expected to improve the sensitivity of measles surveillance by employing a broader suspect case definition requiring detailed active investigation of all suspected cases. As countries approach the elimination threshold, it will be critical to investigate each confirmed case of measles to determine sources of infection and reasons for lack of immunity. It will also be crucial to collect throat swab samples for viral genotyping, in addition to the serum specimens collected for serological confirmation. Elimination standard surveillance requires robust surveillance and laboratory capacity, as well timely and intensive investigation of sporadic as well as outbreak cases and is expected to be more costly to implement [9].

The sensitivity of measles surveillance and the quality of data generated is critically important in the verification process. Without adequate surveillance sensitivity consistently attaining the performance indicators including characterization of circulating viral genotypes, it is difficult to generate evidence required to verify elimination. For example, NVCs in some countries in the WHO European region have been unable to determine whether disease transmission remained endemic or was interrupted. Reasons included inadequate surveillance systems with low sensitivity producing incomplete surveillance data that could not be clearly interpreted to demonstrate evidence in support of elimination; as well as inadequate or incomplete evidence of population immunity [10]. In order to improve the quality of NVC documentation, Italy implemented subnational level assessment of progress and subnational compliance with the elimination criteria [11].

The measles verification framework shares similarities with the polio-free certification process. For a region to be certified polio free, the Regional Polio Certification Commission (RCC) will consider the following: i) the absence of wild poliovirus for at least 3 consecutive years from any source, in the presence of high quality, certification-standard AFP surveillance; ii) high routine immunization coverage with the third dose of oral polio vaccine (OPV3); iii) the completion of phase 1 poliovirus containment activities; iv) country readiness to respond to any poliovirus importation; v) the presence of a functional National Certification Committee to critically review, endorse and submit complete documentation to the RCC [12–14].

## The establishment of measles verification procedures and structures in the African Region

In March 2017, the WHO Regional office for Africa developed and disseminated regional guidelines for the verification of measles elimination. Official communication was sent from the WHO regional office to 32 of the 47 countries in the region between May 2017 and February 2019, requesting them to establish an NVC and to commence the work of documenting progress towards elimination according to the regional guidelines and documentation template. WHO offered technical and financial assistance to establish NVCs. Not all countries were invited to establish NVC at the same time for several reasons. First, there is a limited number of technical staff from the WHO regional and sub-regional offices available to conduct briefings of the newly established NVCs. Second, countries were selected based on their relative progress towards the measles elimination targets in those countries nearing the elimination targets, and the potential advocacy value of NVCs to advance the implementation of elimination strategies in countries requiring significant improvement in their national immunization performance to advance towards measles elimination. A staged implementation of NVCs also allowed lessons to be learned from the initial country experiences.

The global framework and guidelines outline the process and requirements for the documentation of measles and rubella/CRS elimination. At present, in the absence of a formal regional goal of rubella/CRS elimination the African regional guidelines are limited to the verification of measles elimination. The regional verification framework, the process and the role of the verification structures was presented and discussed in various annual meetings of national immunization program managers’ in 2018 and 2019. Additionally, an initial workshop was conducted in March 2018 to orient the members of the RVC. The first five countries to submit documentation of progress to the RVC were reviewed in May 2019. The status of establishment and functionality of NVCs as of April 2019 is summarized in Table 1.

**Table 1:** Current status of establishment and functionality of NVCs in the African region, April 2019

Current status with NVC establishment	Countries
Countries requested to establish NVC; NVC not yet established	Benin, Botswana, Burkina Faso, Cameroon, Chad, Democratic Republic of Congo, Cote d’Ivoire, Eritrea, Ethiopia, Kenya, Lesotho, Liberia, Malawi, Mauritania, Mauritius, Mozambique, Sierra Leone, Togo
NVC established but not yet briefed	Cape Verde, Gambia, Niger
NVC established and briefed	Algeria, Eswatini, Senegal
NVC established, briefed and documentation started	São Tomé & Principe, Tanzania, Uganda
NVC submitted initial progress report to RVC	Ghana, Nigeria, Rwanda, Seychelles, Zimbabwe

## Challenges

Despite the creation of NVCs and the organization of briefings for the NVC members, as of May 2019, only 8 countries in the region have begun to document progress toward elimination. A summary of the most common impediments in establishing NVCs and documenting country progress is detailed below.

### Challenges with the establishment of NVCs

***Inadequate awareness:*** national immunization program managers do not fully understand the purpose and function of NVCs. The justification and terms of reference for NVCs as well as the process of documentation of progress were presented in annual program meetings. However, misconceptions persist including the opinion that countries need to establish NVCs only when they get closer to claiming measles elimination status. Actually, the process of documenting progress with NVC oversight is expected to help weak performing countries to critically review their data, improve program performance and benefit from the advocacy of the NVC with national authorities and partners.

***Multiplicity of committees:*** discussions with various national immunization program managers have revealed concern about the existing multiplicity of national committees and advisory groups to support immunization. There is a limited pool of dedicated and available scientists and experts to engage in such voluntary work, especially in the smaller countries. WHO AFRO has indicated that countries may opt to utilize the expertise in the current national polio certification committees for the purpose of measles verification if practical. However, it is necessary to amend the terms of reference and nomenclature of the committee and conduct a technical briefing of the committee members.

***Availability of technical experts:*** WHO recommends that the membership of NVCs include specialists from various fields (clinicians, laboratory experts, epidemiologists, etc.) who will participate in the committee on a voluntary basis. However, in smaller countries, the available pool of high-level expertise from academic, research and clinical settings is often limited. In addition, available experts often have multiple professional responsibilities and engagements, and often are already engaged as members of NITAG, National Polio expert committee, national polio certification committee, or national polio containment taskforces.

***Prioritization of verification work:*** national immunization program staff handle numerous programmatic priorities and are fully engaged in a multitude of activities, including the development of annual and multiannual plans, development of GAVI application documentations, new vaccine introductions, SIAs, program assessments and appraisals, outbreak response activities and responding to the effects of civil conflict and natural emergencies. The NVCs require the attention, time and dedicated support of the national immunization program team, and the WHO country office immunization team to be fully functional.

***Inadequate human resources at regional level:*** there is a limitation of program staff in the WHO regional and sub-regional offices responsible for the overall coordination of measles and rubella elimination work. For this reason, it was not possible to quickly scale up and establish NVCs in multiple countries, conduct initial briefings and provide continuous to support the work of the NVC including associated work with data management and regular follow-up of the verification documentation at country level.

***Inadequate funding to support country level NVC activities:*** WHO provides catalytic funding for the establishment and functioning of the NVCs at country level. These funds cover costs related to the organization of technical meetings, joint working sessions to analyze data and prepare the country progress reports, supply stationery material and cover costs related to in-country travel when necessary. Currently, the WHO Regional office has limited committed funding to support NVC activities, requiring prioritization in the support to countries to establish NVCs.

### Challenges with the implementation of elimination strategies and data quality

***Data quality:*** in many countries in the African region, vaccination administrative data overestimates the levels of population immunity as compared to survey and WHO UNICEF estimates of coverage. This discrepancy also exists in data at the subnational level. As a result, unless there are recent coverage surveys done to estimate subnational levels of coverage, it is often difficult to assemble accurate information regarding population immunity levels [15, 16]. The measles strategic planning (MSP) tool can provide national measles immunity profiles across multiple age cohorts to better estimate population immunity. However, the utility of the MSP tool is limited because it cannot consider subnational level coverage data [17].

***Incomplete implementation of measles elimination strategies:*** as of April 2019, only 27 of the 47 countries in the region have introduced MCV2 in their routine immunization schedule. For countries having MCV2 for more than 3-5 years, the drop-out rate between MCV1 and MCV2 is more than 10% in 17 out of the 26 countries for 2017. This is a major programmatic weakness having a bearing on the documentation of one of the lines of evidence [4, 18]. In the case of large countries, like Nigeria or Ethiopia, there is a substantial difference at subnational levels in the implementation of elimination strategies that results in differential levels of progress towards elimination, which can be masked when viewed at the national level.

***Surveillance funding gaps:*** forty four out of 47 countries in WHO African region have been implementing measles case based surveillance since at least 2006, with the support of a network of national and regional referral measles serological laboratories. However, over the past five years, the quality of case-based surveillance has not been improving across the region despite the fact that countries are approaching the 2020 target date for elimination [19]. This is compounded by coordination challenges when disease surveillance and immunization are under different divisions within Ministries of Health. Most countries do not allocate adequate funding to support measles surveillance activities. Mobilizing adequate funding is critical to scale up surveillance performance and to implement elimination-standard surveillance when nearing the elimination targets.

***Stock out of lab test kits:*** the regional serological measles laboratory network consists of 49 national and subnational laboratories in 44 countries across the region. The network is supported by WHO to implement standardized testing methods, utilizes similar test kits and is supported with periodic external quality assurance and accreditation exercises. In the period from 2015 to 2017, nearly all the laboratories in the regional measles laboratory network experienced prolonged periods of stock-out of laboratory test kits as a result of delays in resupplying attributed to inadequate funding. This has seriously limited the surveillance system’s sensitivity and its ability to generate high quality information for the purpose of verification [20].

***Lack of genotypic data:*** despite the availability of services in the regional reference laboratories to perform molecular characterization of measles and rubella viruses, many countries have not yet made full use of this opportunity and therefore lack the baseline data required to assess endemic transmission patterns and distinguish them from importations that is important for the verification of elimination [20].

***Inadequate data on CRS occurrence:*** CRS sentinel surveillance is established in only 9 countries across the region. However, several countries have some documentation from retrospective case reviews. CRS is often not recognized commonly as a clinical condition, and requires more specialized clinical skills and diagnostic equipment for initial case detection, there is lack of adequate documentation at country level [5].

## Opportunities and successes

***Previous experience with polio certification:*** countries across the region already have extensive experience with the process of preparing polio eradication progress reports and national certification documentation. The lessons from African regional certification of polio eradication are being utilized to ensure that the NVCs and the RVC establish robust processes from the outset [13, 14].

***Functional regional commission:*** the regional director of the WHO African regional office has officially nominated the members of the Regional Verification Commission. The commission received its introductory briefing in March 2018. The second RVC meeting in May 2019 was used to review the progress reports from 5 countries. The RVC review of country documentation has helped to identify the strengths and weaknesses in country programs with regards to documenting the lines of evidence. The lessons from this exercise will be used to assist other countries, to use the opportunity to critically review their program data and the implementation of measles elimination strategies.

***Advocacy value of verification committees:*** while the main objective of the NVCs and the RVC is to support countries to develop high quality documentation of progress towards elimination along the five lines of evidence, the terms of reference of the NVCs were designed to include advocacy as one of the key functions in their respective countries and at regional level for the RVC. The members of the committees are prominent clinicians, academicians and researchers whose professional reputations can garner support, visibility and influence policy makers in favor of measles elimination.

***External technical assistance:*** to advance the work of verification of measles elimination, the WHO regional office received support from the US Centers for Disease Control (CDC) to complete a detailed analysis of programmatic data in Seychelles and Rwanda to compile their initial documentation submitted to the RVC. This work has helped to critically examine data quality and availability issues, as well as to refine the documentation template.

***Country by country verification:*** the verification of measles elimination is assessed country-by-country, unlike the polio eradication program, where certification is done only on a regional basis. Such a country-focused approach gives high performing countries the opportunity to get official recognition for their progress and motivates others to strive to attain the elimination targets. In addition, when countries are presenting their progress report to the Regional Verification Commission, other NVCs and national immunization program managers are invited to participate and learn from the other country experiences.

## Recommendations

In order to address these challenges and strengthen the ability of NVCs to document progress towards measles elimination, the following priority actions will need to be taken at regional and country levels.

***Raise awareness:*** utilizing all opportunities to communicate to the national authorities and immunization program managers regarding the value NVCs can provide to assist countries with documenting progress towards measles elimination and advocating for better government ownership and partner support.

***Document and disseminate progress:*** scaling-up the documentation of progress towards measles elimination among the high performing countries to help them verify elimination as early as possible and to document the advocacy work of NVCs.

***Technical assistance:*** develop a regional pool of consultants that can assist countries in preparing the initial documentation of progress for review by NVCs.

***Capacity building:*** WHO will continue to build the technical capacity and broader programmatic understanding of NVC members by engaging them as participants in immunization program technical meetings.

***Networking:*** create opportunities and platforms for better networking and experience sharing among NVCs.

***Funding:*** WHO and partners to allocate predictable funding to support the work of NVCs.

***Sub-national documentation:*** in large countries, explore the possibility of NVCs monitoring and documenting progress toward measles elimination sub-nationally by province/State/Region level with their own documentation exercise. This will be a resource intensive exercise to be done in one or two countries, making sure not to burden national programs and in such a way as to carefully document lessons.

## Competing interests

The authors declare no competing interests.
